# Bibliography

**Published:** 1891-07

**Authors:** 


### BIBLIOGRAPHY.

W. D. Miller, A.B., Ph.D., M.D., D.D.S. By W. C. Barrett, M.D., Buffalo, N. Y.

This is a brief but an interesting account of the active life of an earnest and untiring investigator. It was originally printed in the *Dominion Dental Journal.* The excellent likeness of Professor Miller, therein given, accompanies this. It seems as though a portion of the narrative might have been omitted, as it relates to personal matters with which the public have nothing to do.

SCHWEIZERISCHE VIERTELJAHRSSCHRIFT FÜR ZAHNHEILKUNDE.

This quarterly journal is published under the charge of the Swiss Odontological Society, and is printed in two languages—French and German. The former is under the management of Professor Dr. C. Redard, of Geneva, and the latter Dr. Theo. Frick, of Zürich. The first number is filled with interesting matter, and under the two able editors will no doubt exert a valuable influence in Switzerland.

J. B. LIPPINCOTT COMPANY announce the eighth edition of H. C. Wood's "Therapeutics" in press, to be "ready for the use of medical students in the autumn." The work has been thoroughly revised and articles brought up to date.

P. BLAKISTON Son & Co. announce for early publication "A Hand-Book of Local Therapeutics." Harrison Allen, M.D., George C. Harlan, M.D., Charles B. Penrose, M.D., Arthur Van Harlingen, M.D., will have charge of the different parts devoted to the Respiratory Passages, Ear, Eye, Skin, etc. Dr. George I. McKelway will describe the pharmaceutical properties of each remedy.

